# Comparing methods of cellular segmentation in prefrontal and primary visual cortices in Alzheimer’s disease

**DOI:** 10.1002/alz.093006

**Published:** 2025-01-03

**Authors:** Alec K McKendell, Elizabeth McDonough, Lisa Lowery, Samantha Abate, Daniel Meyer, Jennifer Luebke, Patrick R Hof, Merina Varghese

**Affiliations:** ^1^ Icahn School of Medicine at Mount Sinai, New York, NY USA; ^2^ GE HealthCare, Niskayuna, NY USA; ^3^ Boston University School of Medicine, Boston, MA USA

## Abstract

**Background:**

In Alzheimer’s disease (AD), specific brain regions become vulnerable to pathology while others remain resilient. New methods of imaging such as highly multiplexed immunofluorescence (MxIF) provide an abundance of spatial information, while analytical techniques like machine learning (ML) can address questions of cellular contributors to this regional vulnerability.

**Method:**

We performed MxIF staining for 26 markers and compared postmortem human samples from an AD‐susceptible brain area, the prefrontal cortex (PFC, Brodmann’s areas 9, 10 or 46) to an AD‐resilient brain area, the primary visual cortex (V1, area 17). Subjects included AD (n = 3, clinical dementia rating CDR 3, Thal stages 3‐4, Braak stages V‐VI), mild cognitive impairment (n = 4, CDR 0.5, Thal stages 1‐3, Braak stages I‐V), and age‐matched healthy controls (n = 5, CDR 0, Thal stages 0‐1, Braak stages I‐II), including both females and males. We first used a custom Fiji plugin that leverages iterative ML to segment the images via DAPI or HUD signals. For the DAPI segmentation, ML subsequently classifies the cell type based on relevant channels. The second method used QuPath to segment exclusively using DAPI signal followed by cell‐type classifications. Segmentations and classifications in QuPath used static thresholds.

**Result:**

Using Fiji for HUD segmentation and filtering by the presence of nuclei, we found a decrease specifically in layer 5 PFC neurons from CDR 0 to CDR 0.5. Surprisingly, this decrease did not hold in QuPath when segmenting first using DAPI. Both methods found an increase in GFAP‐expressing astrocytes primarily in layer 1. However, the pan‐astrocyte marker ALDH1L1 showed an inverse relationship, with higher density found in layers 2 through 6. Both findings held true regardless of brain region or CDR. Finally, in addition to the white matter, both methods found an increase in oligodendrocytes (MBP+, MBP+/HUD+) within layer 4 of V1 and driven by CDR 0.5.

**Conclusion:**

Comparisons between the image analysis modalities demonstrate that the method by which cells are segmented and classified can impact the interpretation of MxIF data. Combining knowledge of disease biology with a deeper understanding of when and how to use these techniques will improve data precision.

